# MMPFNet: A Novel Lightweight Road Target Detection Method of FMCW Radar Based on Hypergraph Mechanism and Attention Enhancement

**DOI:** 10.3390/s26041291

**Published:** 2026-02-16

**Authors:** Dongdong Huang, Dawei Xu, Yongjie Zhai

**Affiliations:** 1 College of Information and Electronic Engineering, Liming Vocational University, Quanzhou 362000, China; 20221041@lmu.edu.cn; 2 Department of Engineering Science, Macau University of Science and Technology, Macau 999078, China; 3 Department of Automation, North China Electric Power University, Baoding 071003, China; zhaiyongjie@ncepu.edu.cn

**Keywords:** target detection, lightweight model, deep learning, Range-Angle map, FMCW radar sensor

## Abstract

Road target detection is a crucial aspect of current research in automotive advanced driver assistance systems and intelligent transportation systems, where accuracy, speed, and lightweight design are key considerations. Compared to various sensors employed in driving assistance systems, millimeter-wave radar offers advantages such as all-weather operation, low hardware cost, strong penetration capability, and the ability to extract rich spatial information about targets. This paper tackles the challenges posed by the characteristics of Range-Angle map data from 77 GHz Frequency-Modulated Continuous Wave radar—namely, non-visible light imagery, abstract representation, rich fine details, and overlapping features. To this end, this paper proposes MMPFNet, a lightweight model based on the hypergraph mechanism with attention enhancement, as an extension of YOLOv13. First, an M-DSC3k2 module is proposed based on the hypergraph mechanism to enhance attention toward small targets. Second, a detection head with a double-bottleneck inverted MBConv-block structure is designed to improve the model’s accuracy and generalization capability. Third, a lightweight PPLConv module is customized to transform the backbone network, enhancing the model’s lightweight design while slightly reducing its accuracy. Considering the differences from traditional visible light datasets, the Focus Expansion-IoU loss function is introduced into the model to focus attention on different regression samples. The MMPFNet model achieves significant improvements in detecting common road targets such as pedestrians, bicycles, cars, and trucks on the Frequency-Modulated Continuous Wave radar Range-Angle dataset compared to the baseline YOLOv13n model: mAP50-95 increases by 16%, precision improves by 6%, and recall rises by 8.7%. MMPFNet is also evaluated on other non-visible light datasets such as CRUW-ONRD and soundprint datasets. Compared to commonly used detection models like FCOS and RetinaNet, MMPFNet achieves significant performance gains, attaining state-of-the-art results.

## 1. Introduction

In recent years, the convergence of traditional automotive manufacturing with energy, information technology, and transportation sectors has become an unstoppable trend. The electrification and intelligentization of vehicles have emerged as the primary direction for future automotive industry development. With the rapid advancement of autonomous driving technology, reliable target detection has become a core element in ensuring safe vehicle operation. The Advanced Driving Assist System (ADAS) serves as the cornerstone of intelligent vehicle implementation. Enhancing its functionality and expanding its application range have become focal points of current research in both academia and industry.

### 1.1. Backgrounds

In ADASs [1,2], when the vehicle computer makes behavioral decisions, it typically requires detecting road targets. This is usually achieved using cameras, lidar, or millimeter-wave radar as sensors, or through fusion schemes for comprehensive decision-making [3,4]. This often necessitates complex real-time data acquisition and fusion solutions, along with high hardware requirements to process composite information for rapid real-time signal processing and decision-making. A millimeter-wave radar [5,6,7] offers distinct advantages over traditional optical technologies. It operates reliably in all weather conditions, unaffected by lighting, rain, snow, or haze. It exhibits high sensitivity to radial target movement and provides three-dimensional spatial information including distance, azimuth, and height. Compared to lidar, a millimeter-wave radar features lower equipment costs, strong penetration capabilities, and maintains high efficiency even in adverse weather conditions. Furthermore, millimeter-wave radar possesses the ability to perform detailed feature extraction on targets. Combined with optimized detection methods in interference-prone environments [8], this capability gives it a distinct advantage in target detection, making it a very active and highly topical research direction in recent years. It holds significant potential to replace lidar and optical sensors as the mainstream sensor in autonomous driving and intelligent transportation systems, carrying substantial scientific significance and broad application prospects.

By preprocessing raw radar echoes, two-dimensional data such as Range-Doppler maps, Range-Angle (RA) maps, and Time-Range maps can be obtained. Leveraging this information, effective methods for suppressing interference are applied to improve metrics such as the signal to interference plus noise ratio (SINR). Subsequently, various neural network tools are employed for feature extraction and recognition, thereby enabling effective target detection. Current mainstream road target detection models and architectures can be broadly categorized into the You Only Look Once (YOLO) series [9,10], Transformer models [11,12], and improved models based on Faster Region-based Convolutional Neural Networks [13,14]. Each category emphasizes different aspects of accuracy, speed, and application scenarios. The YOLO model architecture, with its flexibility, allows for improvements in its backbone network, detection heads, and target scene requirements. It can significantly enhance the accuracy and robustness of target detection under conditions demanding high real-time performance and limited hardware computing power. Consequently, it has become a crucial technical foundation in the fields of intelligent transportation and autonomous driving.

### 1.2. Main Contributions

To further enhance the variety and accuracy of road target detection, optimize the dataset processing procedure, and improve the feasibility of edge deployment. We employ a 77 GHz Frequency-Modulated Continuous Wave (FMCW) radar to collect a dataset of common road targets. We generate RA video and subject each frame’s RA map to post-processing: Non-Coherent Integration, Scene Adaptive-Constant False Alarm Rate Detector, peak search, and Multiple Signal Classification super-resolution. This process generates single-frame RA maps that meet the input requirements of the subsequent detection model, thereby establishing the FMCW radar RA map dataset. Aiming at the characteristics of RA maps such as non-visible light, data abstraction, easy to confuse and overlap, this paper proposes a lightweight detection model based on hypergraph mechanism and attention enhancement, MMPFNet. Building upon hypergraph mechanism, the model embeds MCAttn into the M-DSC3k2 module of depthwise separable convolution, which improves the attention for small targets, especially pedestrians in FMCW radar RA maps. We design a dual-bottleneck inverted structure MBConv-block detection head, enhancing the model’s accuracy in detecting road target categories and expanding its generalization capabilities. To further adapt to edge deployments with moderate to low hardware capabilities, the MMPFNet model customizes a lightweight PPLConv module to modify the backbone network. With this approach, a slight reduction in accuracy is observed in exchange for a substantial improvement in model efficiency. Finally, considering the differences from traditional visible-light datasets, the model incorporates the Focus Expansion-IoU loss function to prioritize attention on diverse regression samples. Through these innovative enhancements, the MMPFNet model achieves a 98.4% detection accuracy on the 77 GHz FMCW radar RA map dataset. Simultaneously, computational efficiency is significantly enhanced, with parameters and GFLOPs substantially reduced compared to baseline models and related visible/non-visible light detection models. This establishes the optimal solution for detecting road target categories (pedestrians, bicycles, cars, trucks) using only RA map features from FMCW radar.

The main contributions of this paper are as follows:

(1) Generation of FMCW Radar RA Map Dataset: To address the complexity of multi-feature processing and the cumbersome nature of model training, we adopt a simplification strategy by utilizing only the feature of RA maps for target detection. Through careful parameter adjustment and combination of the modules in the single-frame RA maps generation process, we construct the FMCW radar RA map dataset. This lays the foundation for developing a model capable of fast, real-time, and high-accuracy detection and classification of road targets.
(2) Design of the MMPFNet Model: Confronted with the fact that most existing models are designed primarily for visible light datasets and exhibit low detection accuracy and high computational complexity, this paper builds upon the hypergraph mechanism of YOLOv13 [15] to propose the M-DSC3k2 module for enhancing attention toward small targets. A double-bottleneck inverted MBConv-block detection head is introduced to enhance detection accuracy and computational efficiency. Furthermore, a PPLConv module is designed to lightweight the backbone network, achieving an effective balance between target detection accuracy and model efficiency. Finally, the Focus Expansion-IoU loss is incorporated into the model architecture to enhance the detector’s adaptability to non-visible light datasets.
(3) DSC3k2 Module with Cross-Scale Feature Enhancement: To address the issues in the baseline, where global average pooling struggles to fully leverage cross-scale correlations and targeted feature extraction and attention for small targets are insufficient, we propose an improved method using the attention-enhanced DSC3k2 module. By incorporating the random sampling pooling operation of the MCAttn module, the DSC3k2 module can process richer feature information, enabling the model to more comprehensively understand targets in RA maps according to their characteristics and thereby enhancing overall feature extraction capability.
(4) Efficient Detection Head with Dual-Bottleneck Inverted Structure MBConv-block: To address the limitations of the baseline model when handling non-visible light datasets, especially its poor performance and low detection rates on the FMCW radar RA map dataset. We propose a novel lightweight MBConv-block detector with dual tracks. This detector significantly enhances computational efficiency and detection accuracy while substantially reducing the total number of parameters. Consequently, it lowers storage requirements and mitigates the risk of overfitting.
(5) Efficient and Lightweight Backbone Convolutional Blocks: Addressing the high computational cost of visible-light models and the low accuracy of lightweight ones, we modify Depthwise Separable Convolution (DepthSepConv)’s activation functions, convolution kernels, and structure to create a custom PPLConv module. Embedded into MMPFNet’s input network, it boosts inference speed with minimal accuracy loss, achieving a better balance between precision and speed.

The remainder of this paper is structured as follows: Section 2 presents the development process and current status of target detection using millimeter-wave radar; Section 3 describes the generation process of the FMCW radar RA map dataset and demonstrates its performance; Section 4 elaborates on the relevant methods involved in MMPFNet; Section 5 details the experimental design and results; Section 6 discusses the experimental results, along with the limitations and future work; Section 7 presents conclusion.

## 2. Related Works

Millimeter-wave radar target detection technology primarily involves preprocessing raw radar echoes to obtain data representations such as Range-Doppler maps, RA maps, and Time-Range maps [16]. The rapid advancement of neural networks has significantly promoted the integration of deep learning techniques with millimeter-wave radar, enhancing detection and classification performance through diverse radar signal representations. Numerous studies have employed neural networks for radar target classification, which can be broadly categorized into the following aspects.

### 2.1. Recurrent Neural Network Based Detection Methods

The work in [17] addresses the issue of fusing multi-domain radar information for human activity detection by proposing a hybrid framework that combines one-dimensional and two-dimensional convolutional neural networks with recurrent neural networks, effectively enhancing detection accuracy. A radar target clustering and classification framework based on recurrent neural networks has been demonstrated to achieve efficient discrimination between stationary and moving targets, showing particular effectiveness for non-rigid pedestrian targets [18]. Furthermore, the study in [19] designs a multi-classifier framework based on a recurrent neural network ensemble, integrating binary strategies and a hybrid feature selection algorithm, which achieves an average classification accuracy of 91.46% for six categories of road targets.

### 2.2. Frameworks Based on Convolutional Neural Networks and Machine Learning

The capability of convolutional neural networks to automatically extract feature data, thereby replacing complex processes in traditional radar signal processing chains, has been demonstrated [20]. A system integrating millimeter-wave radar with deep neural networks is capable of classifying and estimating the distance to pedestrian and animal targets, while also offering improved resistance to spoofing attacks [21]. The work in [22] designs four radar data classification models and proposes a machine learning-based framework that enhances robustness and accuracy in complex environments. A convolutional neural network-based framework introduced in [23] overcomes the drawbacks of conventional constant false alarm rate (CFAR) detection in high-resolution settings through an overlapping detection mechanism, thereby improving both detection performance and false alarm control. The study in [24] proposes a method based on root radar cross section and support vector machines, achieving 92% accuracy in classifying pedestrians, vehicles, and cyclists, though it does not distinguish automobiles. A FMCW radar classification method combining kinematic and geometric features with a multilayer perceptron has also been investigated [25], achieving 90.3% accuracy at the cost of relatively long processing times.

### 2.3. Multi-Representation and Hybrid Strategies

A hybrid approach leverages deep convolutional neural networks with RA map inputs together with a random forest classifier using grid map features, with integration leading to improved accuracy [26]. By integrating YOLO with an support vector machine, a method based on radar echo range-bearing maps achieves nearly 93% accuracy in vehicle-pedestrian classification [27]. The approach in [28] employs time-frequency maps as input for classifying vehicles, pedestrians, and bicycles, comparing three network architectures and demonstrating high accuracy and low latency but poor generalization. Efficient classification of vulnerable road users under single-frame measurements is achieved using 3D range-Doppler-angle power spectrum data with convolutional neural networks, though cross-scene generalization remains limited [29]. Subsequent work proposes a YOLO-based system using Range-Angle-Doppler maps, achieving 70.64% accuracy and real-time detection on measured data [30].

### 2.4. YOLO Series and Applications

For target detection, YOLO models offer a more favorable speed-accuracy balance compared to methods based primarily on recurrent or convolutional neural networks [31]. Different versions of YOLO exhibit variations in design, improvement strategies, and performance, with distinct explorations in lightweight and real-time optimization [32]. YOLO models have been extensively applied in medical imaging [33,34], industrial manufacturing [35,36], agriculture and remote sensing [37,38], and autonomous driving [39,40]. The potential of YOLOv5 in autonomous driving is explored in [41], while improvement and optimization methods for the model are investigated in [42]. The work in [43] proposes a lightweight YOLO design enabling real-time detection on in-vehicle embedded devices. Zhao et al. provide a comprehensive overview of deep learning-based object detection, detailing the evolution of mainstream frameworks and identifying key challenges such as small target detection [44]. This limitation directly motivates our research focus on improving detection performance in such scenarios. However, most existing studies are conducted on visible light image datasets, leaving significant room for improvement in detection accuracy, computational speed, and lightweight design for models dealing with non-visible light feature maps and specific scenario-based target detection.

The aforementioned studies provide valuable insights into radar signal processing and road target detection. While existing approaches demonstrate certain optimizations in model application and data handling, several challenges remain. These include simplifying complex data processing workflows, expanding the variety and number of detectable road targets, and improving overall detection accuracy. Furthermore, achieving real-time processing of FMCW radar data streams with low-cost hardware, along with advancing system miniaturization and compatibility, constitutes a set of open challenges. Addressing these aspects forms a key motivation for the work presented in this paper.

## 3. Dataset Generation and Optimization

### 3.1. Signal Processing for FMCW Radar

**Figure 1:**
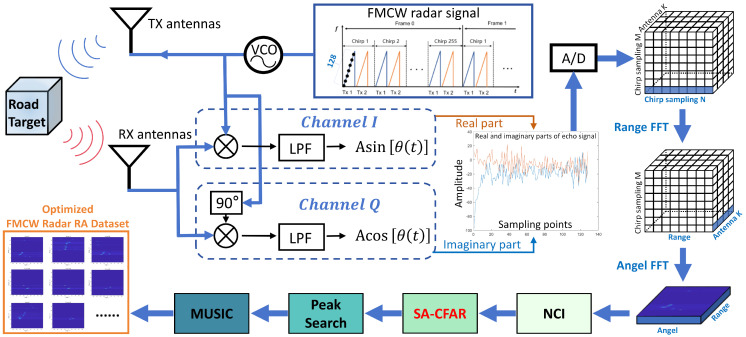
FMCW radar signal processing and optimization workflow.

This paper employs road target radar data collected using Texas Instruments’ AWR1843 radar board. Through FMCW radar signal processing, initial Radar Range-Angle (RA) video is obtained. Subsequently, each RA map frame from the video is adjusted and processed through a pipeline of Non-Coherent Integration (NCI), Scene Adaptive-Constant False Alarm Rate Detector (SA-CFAR), Peak Search, and MUSIC super-resolution. This yields optimized single-frame RA maps suitable for MMPFNet. The overall processing workflow to form the final FMCW Radar RA map dataset is illustrated in Figure 1.

The signal processing workflow is described using a single-frame of RA map from the RA video stream as an example. The specific processing steps are as follows.

#### 3.1.1. Radar Data Acquisition

**Figure 2:**
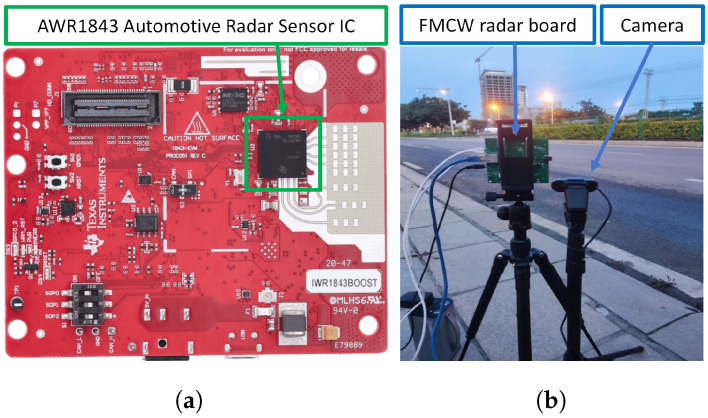
FMCW radar board and data acquisition scenario.

Data acquisition utilizes Texas Instruments’ AWR1843 FMCW radar. The radar acquisition board is shown in Figure 2a, while the actual acquisition scenario is depicted in Figure 2b. An additional camera was installed to capture corresponding RA video footage, serving as an auxiliary tool for subsequent data analysis.

The electromagnetic frequency band of the FMCW radar is 77 GHz. For each radar frame, its raw data (*.mat) has four dimensions: ADC samples (128), linear frequency modulation (255), receive channels (4), and transmit channels (2). All transmitters employ time-division multiplexing (TDM) arrangement, meaning linear frequency modulation signals are transmitted sequentially. The relationships between signal parameters are illustrated in Figure 1 under “FMCW radar signal”.

Through TDM, two transmitters and four receivers form a 1×8 MIMO array, whose transmitted signal FMCW expression is:

(1) $$s_{tx}\left(t\right)=A\cos\left(2\pi \left(f_{0}t+\frac{1}{2}kt^{2}\right)\right)$$

where $f_{0}$ is the starting frequency, and k=B/T is the chirp rate (*B* is the bandwidth, and *T* is the pulse width).

For an echo delay of Δt=2R/c, the received signal expression is:

(2) $$s_{\mathrm{rx}}\left(t\right)=A^{\prime }\cos\left(2\pi \left(f_{0}\left(t-\Delta t\right)+\frac{1}{2}k{\left(t-\Delta t\right)}^{2}\right)\right)$$

#### 3.1.2. Real and Imaginary Parts Processing of Echo Signals

To obtain sufficient information for phase determination, the received signal is split into two channels. We abbreviate Equation (2) as $s_{\mathrm{rx}}\left(t\right)=A\sin\left(\Omega t+\theta \left(t\right)\right)$, and the processing steps for the real and imaginary parts of the echo signal are shown in the blue dashed section of Figure 1.

In Figure 1, the *I* channel is the in-phase channel, and the *Q* channel is the quadrature channel. The mixed signal in the in-phase *I* channel is the local oscillator carrier signal, and the measurement yields the cosine value of the phase modulation component θ(t). The mixed signal in the quadrature *Q* channel is the quadrature carrier signal, yielding the sine value of the phase modulation component θ(t). Signal processing treats the *I* channel signal as the real part and the *Q* channel signal as the imaginary part to form a complex signal, represented as shown in Equation (3). Through this step, the angle information of the echo signal is obtained, given by

(3) $$s\left(t\right)=I\left(t\right)+jQ\left(t\right)=Ae^{j(2\pi f_{\mathrm{beat}}t+\phi )}$$

where $f_{\mathrm{beat}}=k\Delta t=\frac{2kR}{c}$ is the frequency of the differential signal. Through this step, we find the echo signal and angle information.

#### 3.1.3. Generation of Preliminary RA Maps

In FMCW radar, the transmit antenna emits *M* chirp signals per frame, with each chirp comprising *N* samples. Simultaneously, K=4 physical receiving antennas capture return signals. After processing the real and imaginary parts of the echo signals and performing AD conversion, the data forms a three-dimensional block of K×M×N. Through Time Division Multiplexing MIMO (TDM-MIMO) processing, these physical channels are synthesized into $K_{virt}=8$ virtual receiving channels for subsequent angle estimation. By applying Range FFT and Angle FFT operations to this data, the target’s range and angle can be extracted. The specific workflow is illustrated on the right side of Figure 1.

#### 3.1.4. Non-Coherent Integration (NCI)

Following the generation of the preliminary RA maps, this paper adopts non-coherent integration (NCI) to enhance the signal energy across multiple channels, thereby improving the signal-to-noise ratio (SNR) of targets within a single frame. As illustrated in Figure 1, this technique sums the amplitude values of RA maps from different virtual receiving channels along the channel dimension, achieving non-coherent superposition of signals at the same moment. This process is confined to a single frame without involving cross-frame temporal accumulation, thus operating on a per-frame basis and introducing no additional temporal latency. The key advantages of this approach include the following:

(1) Enhanced Target Saliency: Multi-channel amplitude summation suppresses random noise, thereby accentuating the energy distribution of targets.
(2) High Computational Efficiency: The process involves only amplitude addition operations, resulting in low complexity suitable for embedded real-time processing.
(3) Adaptability to Dynamic Scenes: It does not rely on phase alignment, demonstrating good robustness to relative motion between targets and the radar.
(4) Compatibility with Subsequent Processing: The resulting 2D energy map can be directly fed into the SA-CFAR detector, providing a cleaner input for peak search and super-resolution angle estimation.

This step significantly boosts the SNR and detectability of targets within a single-frame RA map, establishing a high-quality image foundation for the subsequent target detection based on the MMPFNet.

#### 3.1.5. Scene Adaptive-Constant False Alarm Rate Detector (SA-CFAR)

**Figure 3:**
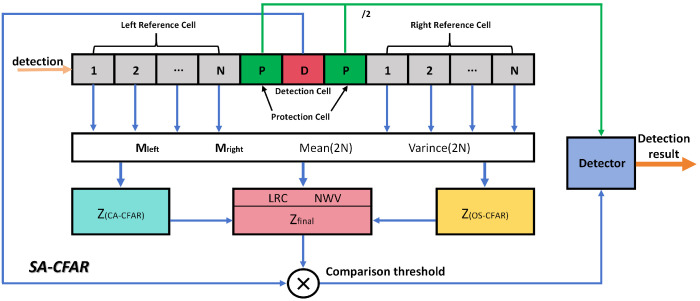
SA-CFAR detector workflow.

Upon completion of the previous step, the radar dataset encompasses both single-target and multi-target fusion scenes. While the conventional CA-CFAR detector offers a high detection probability in single-target scenes with uniform clutter, its performance degrades significantly in scenes with numerous closely spaced targets. Conversely, the OS-CFAR algorithm exhibits superior robustness in such non-uniform, multi-target fusion scenes but at the cost of higher computational complexity. To address this trade-off, we propose a Scene Adaptive-Constant False Alarm Rate Detector (SA-CFAR), which adaptively adjusts its processing strategy based on the scene characteristics, thereby achieving an optimal balance between detection performance and computational efficiency for both single-target and multi-target scenes. The detailed architecture of the proposed SA-CFAR detector is depicted in Figure 3.

In Figure 3, for each detection cell, rapid feature extraction is performed on the reference windows comprising N cells to its left and N cells to its right. These extracted features serve to quantify the uniformity and complexity of the local environment.

The expression for the Left/Right Contrast (LRC), which quantifies the power difference between the left and right reference windows, is as follows:

(4) $$\mathrm{LRC}=\frac{\max(M_{\mathrm{left}},\;M_{\mathrm{right}})}{\min(M_{\mathrm{left}},\;M_{\mathrm{right}})}$$

Among these, $M_{\mathrm{left}}$ and $M_{\mathrm{right}}$ represent the average signal power of the left N cells and the right N cells, respectively.

When LRC approaches 1, it indicates that the background noise power levels on both sides of the Detection Cell are consistent, indicating a uniform region. When this value is significantly greater than 1 (e.g., >2), it strongly suggests that detection is near a clutter edge. LRC is extremely sensitive to clutter edges and serves as a key indicator for distinguishing single-target from multi-target scenes.

The calculation expression for the Normalized Window Variance (NWV) is given as follows:

(5) $$\mathrm{NWV}=\frac{\mathrm{Variance}(2N)}{\mathrm{Mean}(2N)}$$

where Mean(2N) represents the average signal power of the 2N reference cells on both sides. Variance(2N) indicates the dispersion of all 2N reference cell power values relative to their mean value, expressed as follows:

(6) $$\mathrm{Variance}\left(2N\right)=\frac{\sum_{i=1}^{2N}{\left[X_{i}-\mathrm{Mean}\left(2N\right)\right]}^{2}}{2N-1}$$

As defined by the formula, NWV inherently eliminates the influence of absolute power levels, thus providing a more reliable measure of data dispersion.

As illustrated in Figure 3, SA-CFAR computes the background power estimates $Z_{\mathrm{ca}}$ and $Z_{\mathrm{os}}$ for CA-CFAR and OS-CFAR, respectively, based on *N* reference cells on each side. Subsequently, a fusion weighting factor *w*, which combines CA-CFAR and OS-CFAR, is introduced with the following expression:

(7) w=exp(−a·(LRC−1))·exp(−b·NWV)

where *a* and *b* are adjustment coefficients.

The final detection threshold $Z_{\mathrm{final}}=w\cdot Z_{\mathrm{ca}}+\left(1-w\right)\cdot Z_{\mathrm{os}}$. The detection threshold $Z_{\mathrm{final}}$ and the power value of the detection cell are fed into the detector, referencing the average values of the left and right protection cells, to derive the detection value. This ultimately achieves scene-adaptive false alarm detection tailored to single-target scenes and multi-target fusion scenes within radar data.

#### 3.1.6. Peak Search

Peak search serves as a pivotal step in single-frame RA maps optimization, bridging the gap between coarse detection and precise target localization. By detecting local maxima and suppressing interference, it refines the coarse detection results from SA-CFAR into precise target location information. This process balances detection sensitivity with false alarm rate, further refining the preliminary candidate target regions provided by SA-CFAR to ensure each target corresponds to only one detection point. In a two-dimensional RA map, the energy value at a given point must simultaneously satisfy:

(8) P(r,θ)>P(r±Δr,θ)andP(r,θ)>P(r,θ±Δθ)

where Δr and Δθ represent the resolution units for distance and angle, respectively.

Since the energy at the peak point must be significantly higher than that at surrounding points, the following conditions must be satisfied:

(9) $$P_{\mathrm{peak}}>\beta \cdot \frac{1}{N}\sum_{n=0}^{N}P_{\;\mathrm{neighbor}}$$

where β is an empirical coefficient, such as 1.5 to 2.0.

This enables us to localize road targets in single-frame RA maps and proceed to the final processing step.

#### 3.1.7. Multiple Signal Classification (MUSIC) Super-Resolution Processing

After locating road target points through peak detection, we employ the MUSIC super-resolution algorithm on the virtual array synthesized from $K_{virt}=8$ channels to enhance angular accuracy. This approach, based on subspace decomposition and noise orthogonality principles, further enhances the accuracy of target detection in single-frame RA maps. The specific process is as follows:

(10) $$P_{\mathrm{MUSIC}}\left(\theta \right)=\frac{1}{a^{H}\left(\theta \right)U_{n}U_{n}^{H}a\left(\theta \right)}$$

The physical significance can be understood as follows: when (θ) approaches the true target angle, the direction vector a(θ) exhibits maximum orthogonality with the noise space $U_{n}$, causing the denominator to approach zero and the spectral value $P_{\mathrm{MUSIC}}\left(\theta \right)$ to peak. This step enhances the angular resolution of FMCW radar in dense target scenes, enabling more precise single-frame RA maps.

Ultimately, an optimized FMCW radar RA map dataset is formed, comprising 3000 RA maps of single-target and multi-target fusion scenes involving pedestrians, bicycles, cars, and trucks.

### 3.2. RA Maps of Various Types of Road Targets

**Figure 4:**
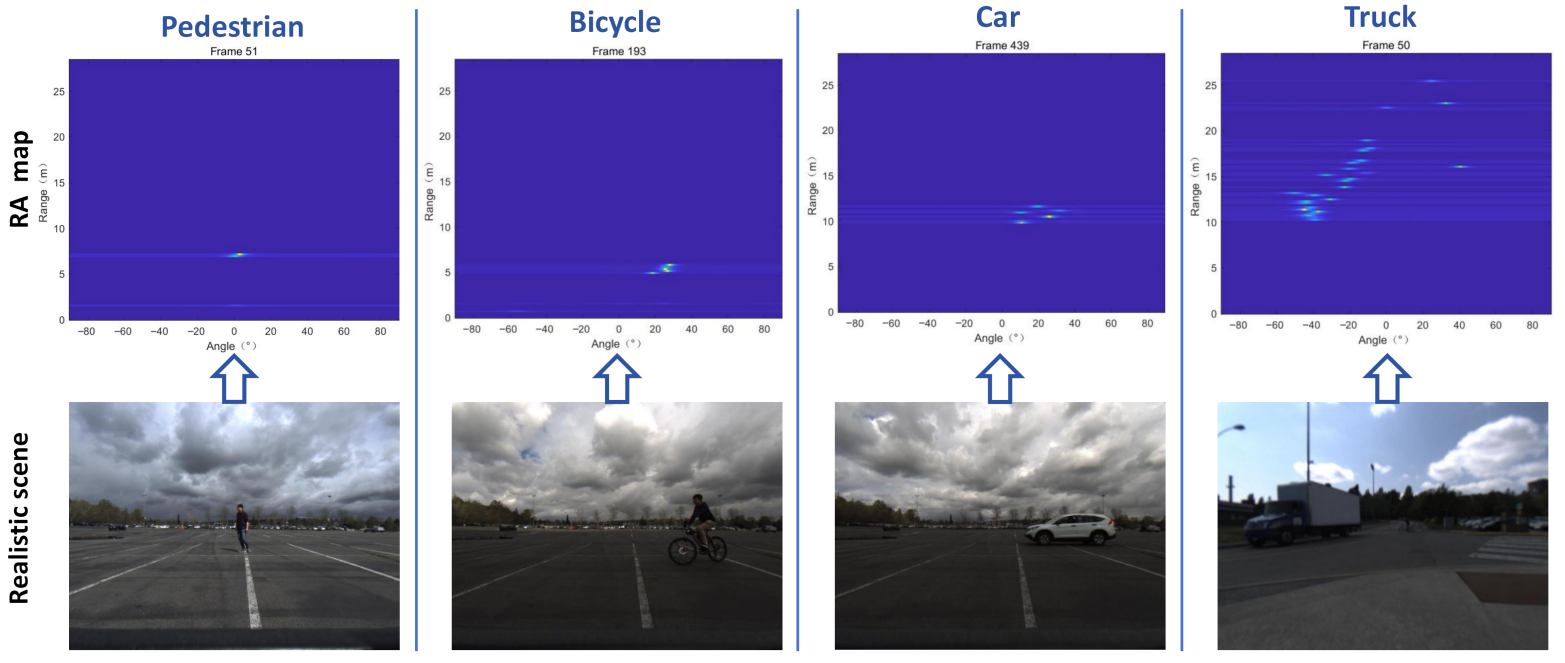
Single-target scenes RA maps.

**Figure 5:**
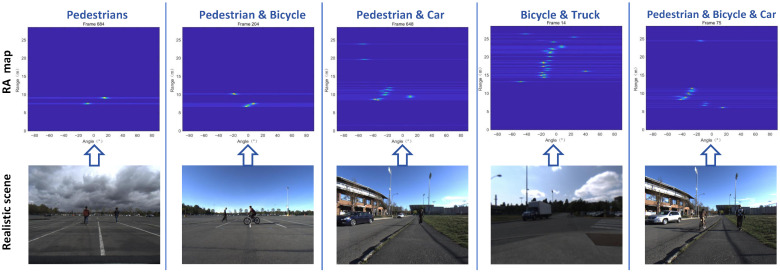
Multi-target fusion scenes RA maps.

By adopting the workflow shown in Figure 1, we obtained optimized RA videos of road targets acquired by the FMCW radar, covering both single-target scenes and multi-target fusion scenes. A representative frame from each scene’s RA video is presented in Figure 4 and Figure 5.

In Figure 4, the first row shows the processed single-frame RA map dataset, where each RA map plots angle on the x-axis and distance on the y-axis. The second row displays the corresponding realistic scene conditions for reference. From the final results, we can observe that RA information for single-target—such as pedestrians, bicycles, cars, and trucks—is accurately represented in the single-frame RA maps of the first row, while also effectively suppressing environmental noise.

In Figure 5, under more complex scenes, the final processing results demonstrate that in fusion scenes involving multiple targets—such as two pedestrians, a pedestrian and a bicycle, a pedestrian and a car, a bicycle and a truck, or a pedestrian, bicycle, and car—each target is clearly represented on the single-frame RA maps while retaining its distinct features.

Taking the “Pedestrian and Bicycle” scene in the second column of Figure 5 as an example, we analyze the angular resolution corresponding to the adopted 2Tx-4Rx TDM-MIMO configuration. The system forms an 8-channel virtual array. After MUSIC super-resolution processing, the angular information of the pedestrian and bicycle targets can be extracted from the RA map. The image width is 640 pixels, and the total angular span is $160^{\circ }$. Measurement shows that the peak separation between the two targets along the angular axis is approximately 30 pixels. The actual angular separation Δθ is calculated as follows:

(11) $$\Delta \theta =\frac{160^{\circ }}{640\;\mathrm{pixels}}\times 30\;\mathrm{pixels}=7.5^{\circ }$$

The results indicate that, after MUSIC super-resolution processing, the system achieves an angular resolution of approximately $7.5^{\circ }$ in practical measurements, which can effectively support the basic discrimination and detection requirements for multi-target fusion scenes at medium-to-short ranges.

The generation and optimization of the FMCW radar RA map dataset have now been completed.

## 4. Methods

### 4.1. Overview of the MMPFNet Architecture

**Figure 6:**
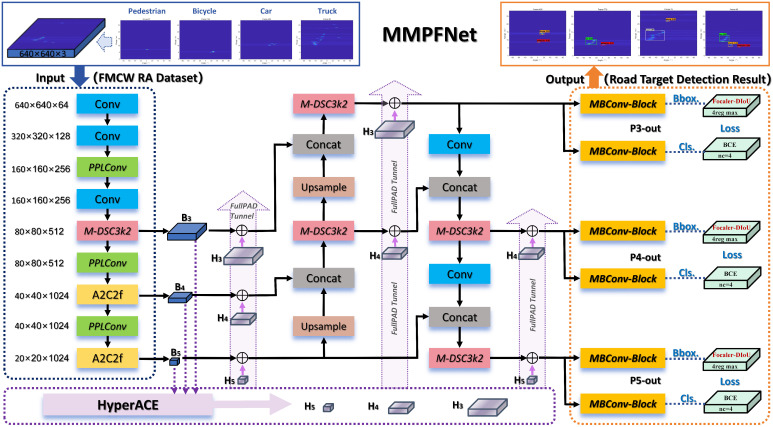
MMPFNet model architecture.

Our proposed MMPFNet is built upon the YOLOv13 baseline and inherits the hypergraph characteristics from its Hypergraph-based Adaptive Correlation Enhancement (HyperACE) mechanism. It demonstrates strong detection performance for non-visible light and small targets in the FMCW radar RA map dataset. Building on this foundation, cross-scale feature refinement is applied to DSC3k2, and an inverted bottleneck structure is introduced for the detection head. This approach elevates the head’s accuracy while advancing its lightweight design in terms of parameters and GFLOPs. Additionally, the Focus Expansion-IoU loss function is introduced to address the impact of varying sample distributions on bounding box regression. Considering subsequent edge deployment, this paper modifies the backbone convolutional network by using DepSepConv as the basic block and performing activation function H-Swish, convolution kernel replacement, and module addition/subtraction operations. This enhances inference speed without significantly compromising accuracy, achieving a better balance between model precision and speed. The overall network architecture is shown in Figure 6 below. Due to space constraints, the detailed structures of M-DSC3k2, MBConv-block, and PPLConv depicted in the figure will be described in the following sections.

### 4.2. M-DSC3k2 Module

**Figure 7:**
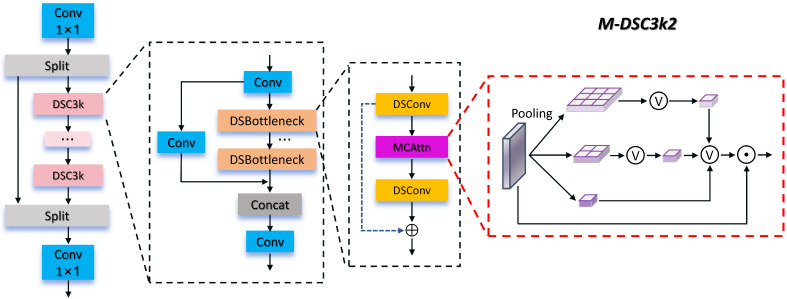
M-DSC3k2 structure.

To enhance the attention to small targets in the FMCW radar RA map dataset, this paper proposes the M-DSC3k2 module, which builds upon the DSC3k2 [15] module by introducing the MCAttn attention module [45] and embedding it into the DSBottleneck of the DSC3k2 module. Its structure is illustrated in Figure 7.

In the DSBottleneck component of M-DSC3k2, a depth-separable convolution first performs depthwise convolution on the input tensor, executing spatial convolution independently for each channel to extract features in the channel dimension. Subsequently, the random sampling pooling operation of the MCAttn module is introduced to generate scale-invariant attention maps. MCAttn captures cross-scale information by randomly selecting attention maps from pooling tensors of three different scales: 3×3, 2×2, and 1×1. The computation of its attention map is shown in Equation (12).

(12) $$A_{m}\left(x\right)=\sum_{i=1}^{n}P_{1}\left(x,i\right)f\left(x,i\right)$$

where *i* denotes the output dimension of the attention map, and f(x,i) represents the average pooling function, which pools features across different scales. The correlation probability $P_{1}\left(x,i\right)$ must satisfy $\sum_{i=1}^{n}P_{1}\left(x,i\right)=1$ and $\prod_{i=1}^{n}P_{1}\left(x,i\right)=0$ to ensure that the generated attention map exhibits scale invariance and strong generalization capabilities. In this study, *n* is set to 3.

The design advantages of M-DSC3k2 are twofold. On one hand, its random selection mechanism for cross-scale attention enables comprehensive capture of semantic information across different scales. This significantly enhances focus on small targets within the dataset, effectively guiding the network to concentrate on and learn subtle features of small targets. Simultaneously, it strengthens the model’s contextual learning capabilities, helping the network better understand the relationship between small targets and their surroundings, as well as the feature variations of small targets themselves at different scale ratios. This ultimately improves the network’s segmentation performance. On the other hand, it inherits the advantages of the DSC3k2 module in depth-separable convolutions. While maintaining network performance, it substantially reduces computational complexity and parameter count, facilitating efficient model deployment and inference.

Therefore, M-DSC3k2 demonstrates superior adaptability to small targets of varying sizes and shapes within FMCW radar RA map dataset. It addresses the limitations of traditional attention mechanisms, which rely on global average pooling and struggle to fully leverage cross-scale correlations. This approach enables more targeted feature extraction and attention modeling tailored to small targets detection tasks.

### 4.3. MBConv-Block Detect Head

**Figure 8:**
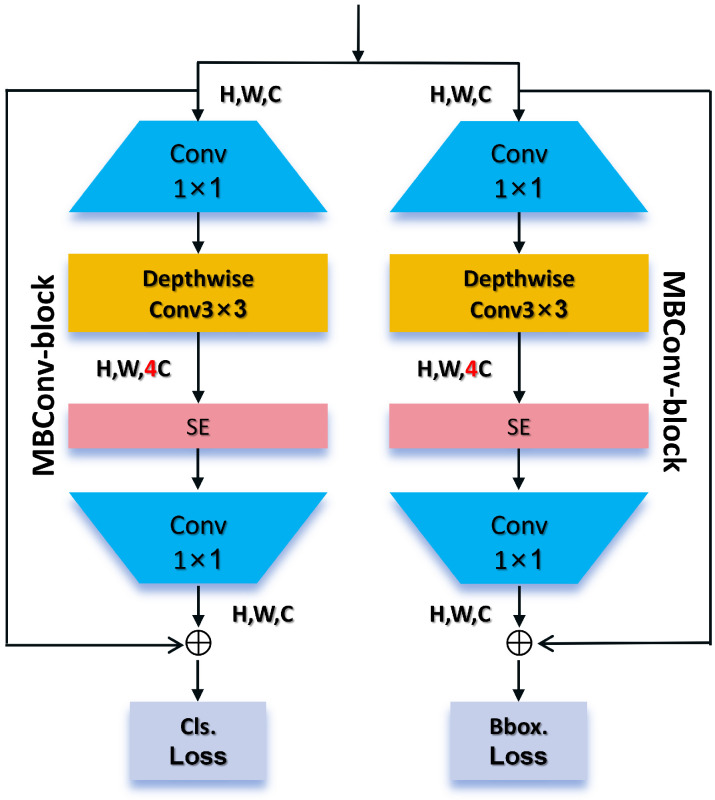
MBConv-block detect head structure.

In the detection head component of MMPFNet, inspired by the mobile inverted bottleneck architecture of EfficientNet [46], we designed a novel double-bottleneck inverted structure MBConv-block detect head. This architecture balances lightweight design, computational efficiency, and detection accuracy, as illustrated in Figure 8.

This detection head innovatively employs a dual-track decoupling design, with two parallel and structurally symmetric tracks dedicated to bounding box regression and target classification, respectively. As shown in Figure 8, the input features $F_{in}\in R^{H\times W\times C_{in}}$ are first fed into two independent branches:

#### 4.3.1. Bounding Box Regression Track

This track focuses on the spatial localization of the target. At its core is an MBConv module that first performs dimension increasing operations on the input features through pointwise convolutions:

(13) $$F_{exp}^{reg}=\mathrm{ReLU}6\left(\mathrm{BN}\left(W_{p1}^{reg}*F_{in}\right)\right)$$

where $W_{p1}^{reg}$ is the unique extended convolution weight for this path. Subsequently, spatial features $F_{dw}^{reg}$ for position refinement are extracted via depth-separable convolution, and adaptive calibration feature channels are utilized through the SE module. The excitation weight $s^{reg}$ is independently computed from the features of this path:

(14) $$s^{reg}=\sigma \left(W_{2}^{reg}\cdot \delta \left(W_{1}^{reg}\cdot z^{reg}\right)\right)$$

Finally, the features are compressed to 4×reg(max) dimensions through the projection layer, producing a distributed representation of the bounding boxes, which is then supervised by the bounding box regression loss.

(15) $$F_{proj}^{reg}=\mathrm{BN}\left(W_{proj}^{reg}*\left(s^{reg}\cdot F_{dw}^{reg}\right)\right)$$

#### 4.3.2. Classification Track

This track focuses on target category classification. It features an MBConv-block architecture symmetrical to the regression track but employs a completely independent parameter set $\left\{W_{p1}^{cls},\;W_{d}^{cls},\;W_{1}^{cls},\;W_{2}^{cls},\;W_{proj}^{cls}\right\}$. Its workflow can be represented as follows:

(16) $$F_{exp}^{cls}=\mathrm{ReLU}6\left(\mathrm{BN}\left(W_{p1}^{cls}*F_{in}\right)\right)$$

(17) $$s^{cls}=\sigma \left(W_{2}^{cls}\cdot \delta \left(W_{1}^{cls}\cdot z^{cls}\right)\right)$$

(18) $$F_{proj}^{cls}=\mathrm{BN}\left(W_{proj}^{cls}*\left(s^{cls}\cdot F_{dw}^{cls}\right)\right)$$

The output dimension of this track is nc=4, representing the number of categories, and it is supervised by the Cls Loss.

This parameter-decoupled dual-track design enables optimization of localization and classification tasks within their respective independent feature transformation spaces, thereby avoiding gradient conflicts and feature interference between tasks. Within each track’s MBConv-block module, the Shortcut connection is activated when input and output dimensions match ($C_{in}=C_{out}$ and spatial stride equals 1): $F_{out}^{path}=F_{in}+F_{proj}^{path}$ where path∈{reg,cls}. This effectively promotes gradient flow and feature reuse within each track.

Through the dual-track operation of the MBConv-block detect head, MMPFNet retains the high-performance advantages of the baseline model’s decoupled head while also gaining the lightweight benefits provided by MBConv-block. Compared with standard convolutions, depthwise separable convolutions significantly reduce both computational complexity and parameter count per track. For a 3×3 convolution, the parameter ratio per track can be estimated as:

(19) $$\frac{{\mathrm{Params}}_{DW}^{path}}{{\mathrm{Params}}_{Std}^{path}}\approx \frac{1}{C_{out}}+\frac{1}{9}$$

The task-specific design and lightweight architecture of the MBConv-block detect head significantly reduce the total number of parameters while enhancing model accuracy and generalization capabilities. This approach lowers storage requirements and mitigates overfitting risks, making MMPFNet highly suitable for deployment in edge deployment with limited computational resources.

### 4.4. PPLConv Module

**Figure 9:**
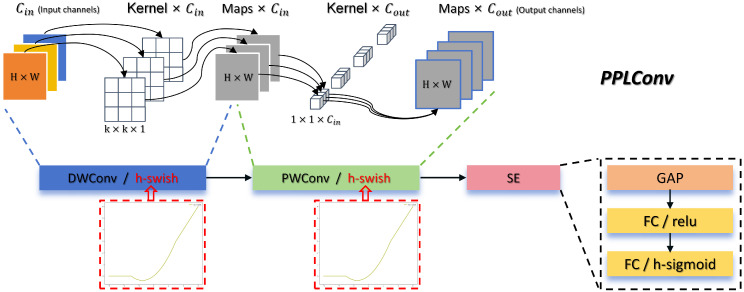
PPLConv structure.

To enable efficient deployment of MMPFNet on lightweight edge devices in the future, we drew inspiration from the design of PPLCNet [47]. By applying some general principles for designing lightweight CNNs, we developed a customized PPLConv core module for MMPFNet’s input network. Its detailed structure is shown in Figure 9.

For an input feature map $F_{\mathrm{in}}\in R^{H\times W\times C_{\mathrm{in}}}$ and a standard convolution kernel $W_{\mathrm{std}}\in R^{k\times k\times C_{\mathrm{in}}\times C_{\mathrm{out}}}$, under normal circumstances, the computational complexity of performing the convolution operation is:

(20) $$FLOP_{s_{std}}=H\times W\times k^{2}\times C_{in}\times C_{out}$$

The PPLConv structure employs MobileNetV1’s DepthSepConv [48] as its basic building block. This structure decomposes standard convolution into two independent operations: depthwise convolution and pointwise convolution, as illustrated by the DWConv and PWConv modules in Figure 9, respectively.

In DWConv, each input channel is independently processed by a k×k×1 two-dimensional convolution kernel, focusing on spatial feature extraction. For $C_{in}$ input channels, its computational cost is:

(21) $$FLOP_{s_{dw}}=H\times W\times k^{2}\times C_{in}$$

In PWConv, a $1\times 1\times C_{\mathrm{in}}$ convolution kernel is employed to perform channel fusion and dimensionality transformation. For $C_{out}$ output channels, its computational cost is:

(22) $$FLOP_{s_{pw}}=H\times W\times C_{in}\times C_{out}$$

The ratio of total computational cost is:

(23) $$\frac{{\mathrm{FLOPs}}_{dw}+{\mathrm{FLOPs}}_{pw}}{{\mathrm{FLOPs}}_{std}}=\frac{1}{C_{out}}+\frac{1}{k^{2}}$$

When the number of output channels $C_{out}$ is large, this structure can achieve a reduction in computational load by more than an order of magnitude.

Additionally, within the PPLConv module, the H-swish activation function is employed after both convolutional operations, replacing the traditional ReLU. H-Swish serves as an efficient approximation of the Swish activation function for mobile devices. As indicated within the red dashed box in Figure 9, it is defined as follows:

(24) $$H\text{-}\mathrm{Swish}\left(x\right)=\frac{\mathrm{ReLU}6(x+3)}{6}$$

Here, ReLU6(z)=min(max(x,0),6), and the function curve is depicted by the red dashed box in Figure 9. This function exhibits smooth curvature near the origin, avoiding the hard zero boundary of ReLU at point x=0. This facilitates improved gradient flow, particularly during the early stages of network training. Its mathematical properties enable the model to learn more refined and information-rich feature representations. This substitution delivers significant accuracy gains at the cost of nearly unchanged inference latency.

Furthermore, PPLConv avoids complex operations such as shortcuts, element-wise addition, or channel concatenation. While these operations offer limited accuracy gains on small models, they significantly reduce inference speed. Furthermore, this simple depthwise separable convolution structure is highly optimized by the Intel Math Kernel Library. This optimization allows it to surpass more complex lightweight modules, such as Inverted Residual Blocks or ShuffleNet blocks, in actual deployment scenarios.

The PPLConv module is embedded into the input convolutional network of MMPFNet to achieve a more lightweight architecture, accepting a marginal compromise in accuracy for substantial gains in efficiency. This lays a solid foundation for efficient deployment on edge devices with limited computational resources.

### 4.5. FE-IoU Loss Function

In target detection tasks, the design of the bounding box regression loss function is crucial to model performance. The baseline model’s original loss function was optimized for its specific dataset (visible light images). The FMCW radar RA map dataset, being non-visible light map dataset, exhibits significant differences in target scale, shape, and distribution complexity. The original loss function failed to effectively distinguish between challenging and simple samples, preventing the model from focusing on more difficult regression samples. This limitation constrained further improvements in detection performance.

To address the aforementioned issues, we introduce the Focus Expansion-IoU (FE-IoU) loss function in MMPFNet. The core concept of this function originates from the “focusing” approach proposed by Focal Loss [49] to tackle class imbalance. We adapt this concept to the bounding box regression domain by innovatively reconstructing the traditional Intersection over Union (IoU) through a linear interval mapping function. This enables dynamic adjustment of attention levels for regression samples of varying quality. The reconstructed IoU is defined as follows:

(25) $$IoU_{R}=\left\{\begin{array}{cc} 0, & IoU<u\\ \frac{IoU-u}{v-u}, & u\leq IoU\leq v\\ 1, & IoU>v \end{array}\right.$$

where IoU represents the raw intersection-over-union ratio between the predicted bounding box and the ground truth bounding box, where [u,v] denotes a predefined threshold interval satisfying 0≤u≤v≤1. This function treats low-quality samples with IoU values below *u* as outliers and assigns them zero weight. High-quality samples exceeding *v* are regarded as easy-to-learn samples and receive unit weight. For medium-quality samples within the interval [u,v], a linear mapping is applied to ensure their gradient weights fall between 0 and 1. Based on this, the fundamental IoU loss is defined as $L_{IoU-R}=1-IoU_{R}$.

However, the single IoU metric cannot fully reflect the regression quality of bounding boxes, especially when gradients vanish for non-overlapping boxes. Therefore, instead of directly using $L_{IoU-R}$ in MMPFNet, we combine it with the EIoU loss [50], which comprehensively considers both overlap area and center point distance, the final FE-IoU loss function:

(26) $$L_{FE\text{-}IoU}=L_{EIoU}+IoU-IoU_{R}$$

(27) $$L_{EIoU}=1-IoU+\frac{\gamma^{2}\left(x,xt\right)}{b^{2}}$$

where *x* and xt denote the center points of the predicted box and the ground truth box, respectively, γ represents the Euclidean distance, and *b* is the diagonal distance of the minimum bounding region encompassing both boxes.

Introducing the FE-IoU loss function: It offers dual advantages:

#### 4.5.1. Adjustable Sample Attention

By tuning thresholds *u* and *v*, we can precisely control the model’s focus on challenging samples (medium IoU). For instance, increasing *u*’s value directs the model to concentrate on learning targets with more difficult localization.

#### 4.5.2. Gradient Property Optimization

The $IoU_{R}$ term acts as a dynamic regulator. For low-quality samples (IoU<u), $IoU_{R}$ equals 0. It interacts with $L_{EIoU}$ but its overall contribution is suppressed due to $IoU_{R}$ being 0. For medium-quality samples, this term produces a smooth gradient, guiding the model toward stable optimization. For high-quality samples (IoU>v), $IoU_{R}$ equals 1, and the loss degenerates into the original $L_{EIoU}$, preventing excessive focus on already well-learned samples.

During the early-stage generation of the RA dataset, the preprocessing pipeline—comprising NCI, SA-CFAR, Peak Search, and MUSIC—effectively suppressed background noise. To ensure the inclusion of all challenging pedestrian samples in training, mitigate the risk of missed detections, and maintain fairness among different types of road targets (i.e., pedestrian samples rarely meet this threshold and thus require continuous optimization, while vehicle samples often exceed it and can be moderately relaxed), we temporarily set u=0.00 and v=0.95. The corresponding effects can be observed in the Ablation Experiments subsection.

After incorporating the FE-IoU loss function, MMPFNet can adaptively adjust the weights of regression samples with varying difficulty levels during training. This effectively enhances the model’s regression capability for challenging samples on the FMCW radar RA map dataset, thereby achieving more robust and accurate performance across diverse complex detection scenes.

## 5. Experiments and Results

### 5.1. Experimental Environment Configuration and Parameters

This experiment was conducted using an Intel^®^ Xeon^®^ Platinum 8269CY CPU@ 2.50 GHz/RAM 32 GB/NVIDIA RTX5000-24Q workstation. The experimental environment was PyTorch 2.0.1 + CUDA 11.8. MMPFNet training parameters were set as follows: batch size = 64, initial learning rate lr0 = 0.001, epochs = 200. To prevent overfitting, patience was set to 30.

The key evaluation metrics examined in this experiment include mAP50, mAP50-95, Precision, and Recall. Specifically, mAP50 and mAP50-95 denote the average precision at an IoU threshold of 0.5 and at each threshold from 0.5 to 0.95 in 0.05 increments, respectively. Precision is defined as the ratio of correct detections to the total number of detected targets, calculated using the following formula:

(28) $$P_{precision}=\frac{TP}{TP+FP}$$

where TP denotes True Positive, while FP denotes False Positive.

Recall is defined as the ratio of correct detections to the total number of ground-truth targets, and its formula is as follows:

(29) $$P_{recall}=\frac{TP}{TP+FN}$$

where TP denotes True Positive, and FN denotes False Negative.

Simultaneously, we also examine the total number of parameters: all weights and biases requiring learning across models under the same dataset and the number of floating-point operations (GFLOPs) required for a single forward inference to validate the feasibility of lightweight optimization.

### 5.2. Experimental Results

**Figure 10:**
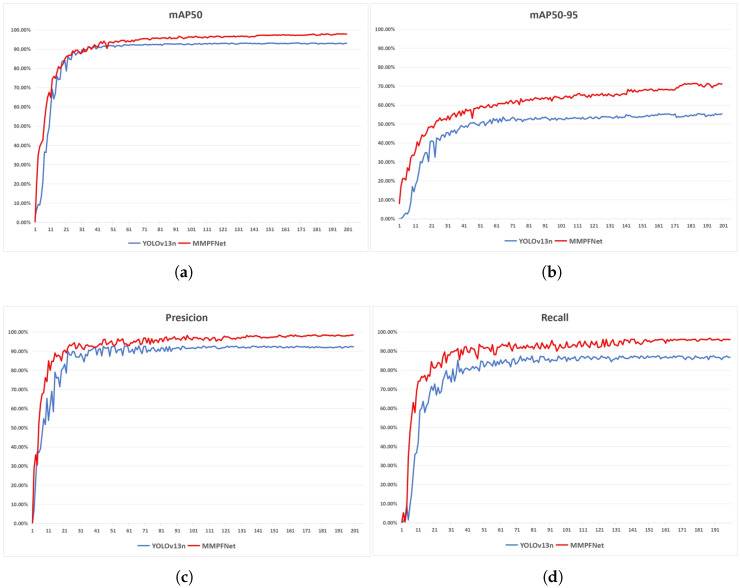
Comparison of parameters between MMPFNet and YOLOv13n.

We compared the experimental results of the MMPFNet model with the YOLOv13n baseline model. To quantify the improvements of MMPFNet over the baseline, both models were trained under identical experimental conditions using the same dataset. The experimental metrics are presented in Table 1 and Figure 10. As shown in Table 1, MMPFNet achieves improvements across all evaluation metrics compared to the baseline model: mAP50 increases by 4.8%, mAP50-95 by 16%, Precision by 6%, and Recall by 8.7%. Figure 10 shows that both models converge around 200 epochs during training. In Figure 10a, their performance remains comparable during the first 30 epochs, but MMPFNet gradually gains an advantage thereafter. Benefiting from the introduction of the FE-IoU loss function, MMPFNet consistently outperforms YOLOv13n in Figure 10b,d, ultimately widening the gap. In Figure 10c, due to the non-visible light nature of the FMCW radar RA map dataset (distinct from traditional image datasets), both models exhibit significant precision fluctuations in the early stages. However, MMPFNet converges faster and achieves better performance than YOLOv13n, attributed to improvements in M-DSC3k2 and the MBConv-block detection head. Table 1 further demonstrates that MMPFNet, through its PPLConv lightweight design, outperforms YOLOv13n across all evaluation metrics while achieving significant reductions in parameters and GFLOPs-45% fewer parameters and 41.9% lower GFLOPs. This enables faster model inference and reduced hardware requirements while maintaining detection accuracy.

**Figure 11:**
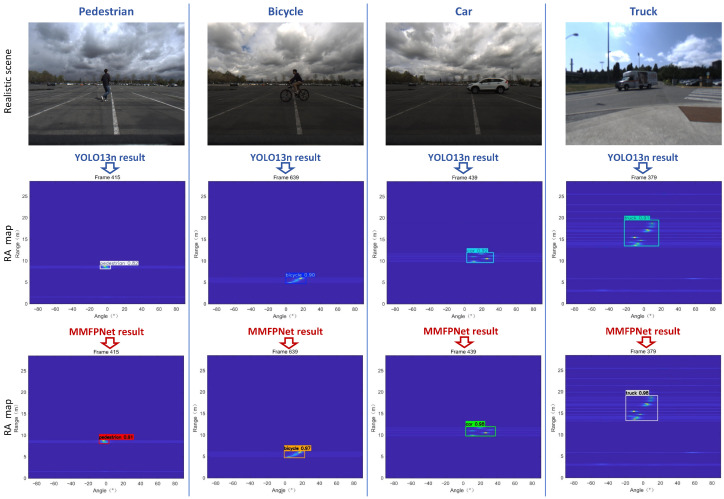
MMPFNet vs. YOLOv13n single-target detection performance comparison.

**Figure 12:**
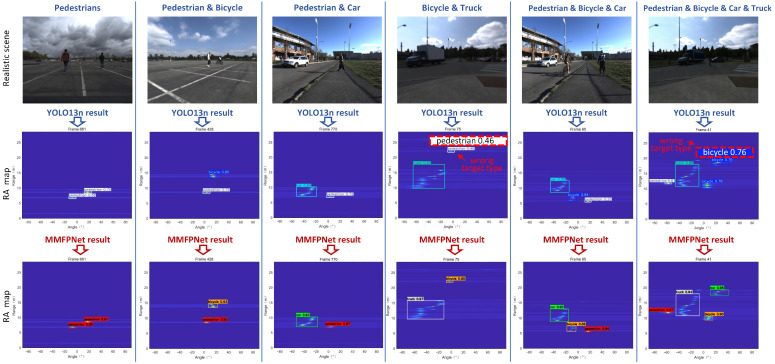
MMPFNet vs. YOLOv13n multi-target fusion scenes detection performance comparison.

The gap in target detection performance between the two models can also be observed in the partial actual results shown in Figure 11 and Figure 12.

Figure 11 presents a comparison of the detection results between the two models for each category of road target. The experimental results clearly demonstrate that MMPFNet achieves higher detection accuracy than the baseline YOLOv13n model for all target categories. This performance gap is particularly pronounced in the detection of small-sized targets such as pedestrians. As evidenced in the first column of Figure 11, where YOLOv13n attains an identification accuracy of 0.82, MMPFNet significantly improves this to 0.91.

Figure 12 presents a comparison of the detection results between the two models in scenes involving the fusion of multiple road target categories. In these more realistic multi-target scenes, differences in attention mechanisms, loss functions, and detection head design lead to a more pronounced gap in detection accuracy compared to the performance observed in single-target scenes. It can be noted that, in the scene shown in the fourth column, the YOLOv13n model incorrectly classifies a bicycle as a pedestrian. Furthermore, in the “Pedestrian & Bicycle & Car & Truck” scene, the YOLOv13n model misidentifies a car as two separate bicycle instances.

It is worth noting that Figure 11 and Figure 12 only showcase partial actual results. Based on our multi-round evaluations, MMPFNet consistently outperforms the baseline YOLOv13n in FMCW radar map detection performance.

### 5.3. Comparative Experiments

#### 5.3.1. Comparison of General Target Detection Models

Through comparative experiments conducted under identical environmental settings using the same dataset, this paper compares the performance of MMPFNet with several other commonly used general target detection models. The specific results are shown in Table 2.

Table 2 reveals that the trained models exhibit varying mAP50 and mAP50-95 metrics on the FMCW RA dataset. Overall, the YOLOv13n model demonstrates improved performance across all metrics compared to v11n and v12n. Regarding Precision and Recall metrics, the YOLO series models v11n, v12n, and v13n show increasing performance with version increments. Specifically, v13n outperforms v11n and v12n in mAP50, mAP50-95, and Precision metrics, shows performance gains with increasing version numbers, while v8n outperforms v11n and v12n in mAP50, mAP50-95, and Precision. v5n slightly surpasses v13n in mAP50-95. The RT-DETR model [51] achieves mid-range performance in mAP50 and mAP50-95 but excels in Recall. Although the Faster R-CNN model lags significantly behind other general-purpose models in mAP50, Precision, and Recall metrics, it substantially outperforms them in mAP50-95. Overall, our proposed MMPFNet outperforms several commonly used target detection models across all evaluation metrics.

This paper also examines two computational metrics: parameters and GFLOPs. As shown in the lower half of Table 2, after training on the same dataset, YOLOv5n, v11n, v12n, and v13n exhibit comparable overall parameters. YOLOv8n demonstrates an increase relative to other versions in the same series, matching the parameters of Faster R-CNN. Notably, however, the RT-DETR model’s parameters are 10 to 13 times greater than those of other models. In terms of GFLOPs, all YOLO variants remain below 10, while Faster R-CNN achieves 15.5. RT-DETR, however, surpasses 100. The proposed MMPFNet demonstrates significant reductions in both parameters and GFLOPs compared to the preceding models. Furthermore, the data in Table 2 and Table 3 serve as a primary basis for selecting YOLOv13n as our baseline.

#### 5.3.2. Comparison of Non-Visible Light Specialized Models

Given that the experimental context involves millimeter-wave radar-based road target detection targeting non-visible light datasets like the FMCW radar RA map dataset, we also selected two common non-visible light target detection models: FCOS (Fully Convolutional One-Stage Object Detection) [52] and RetinaNet [53] for experimentation. FCOS primarily targets non-visible light data types like medical imaging and remote sensing, typically applied in scenarios requiring precise segmentation or localization, such as organ and vessel detection. RetinaNet primarily targets non-visible light data types such as infrared, X-ray, and remote sensing, and is typically applied in scenes involving small target detection and moderate target counts. By referencing the testing methodologies for millimeter-wave radar data in [54,55], we conducted evaluations using two datasets: the CRUW-ONRD dataset and the FMCW radar RA map dataset presented in this paper. The specific results are shown in Table 3. From Table 3, we can see that the parameters of mAP50, mAP50-95, Precision and Recall of MMPFNet on CRUW-ONRD dataset are much better than those of FCOS and RetinaNet with ResNet-101 as backbone. The detection performance of MMPFNet on the CRUW-ONRD dataset is illustrated in Figure 14.

**Figure 13:**
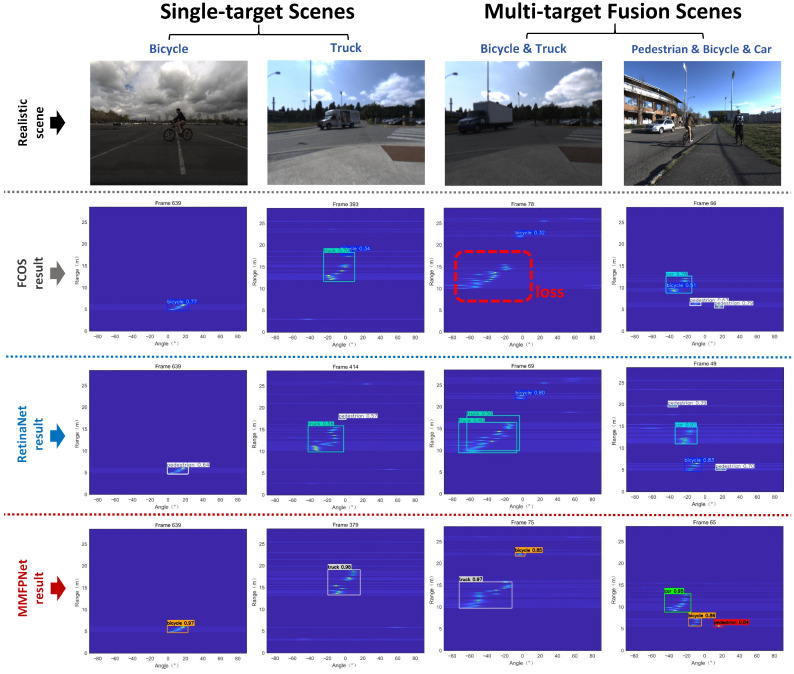
Performance comparison of FCOS, RetinaNet and MMPFNet on the FMCW radar RA map dataset.

In the FMCW radar RA map dataset test results, the performance gap between MMPFNet and FCOS and RetinaNet has narrowed. The detection results of the three models are compared as shown in Figure 13.

From Figure 13, it can be observed that both the FCOS and RetinaNet models suffer from issues such as decreased detection rates, multiple false detections, missed detections, and incorrect target category classifications. For instance, in single-target scenes (e.g., the “Bicycle” scene shown in the first column), the detection accuracy of FCOS (second row) decreases significantly compared to MMPFNet (fourth row), while RetinaNet (third row) misclassifies the target. In scenes rich with features, such as the single-target “Truck” scene, FCOS erroneously detects an additional bicycle, whereas RetinaNet produces a false positive pedestrian detection. In multi-target fusion scenes like “Bicycle & Truck” (third column), FCOS fails to detect the primary target (the truck), and RetinaNet generates an extra false truck detection. In the “Pedestrian & Bicycle & Car” scene (fourth column), FCOS misclassifies the bicycle as a pedestrian and splits the car into two separate detections (a car and a bicycle). Although RetinaNet correctly identifies the pedestrian, bicycle, and car categories, its overall precision declines, and it also produces one additional false pedestrian detection.

Our preliminary analysis indicates that FCOS’s anchor-free frame design avoids sensitivity to anchor frame hyperparameters, simplifies the process, and is effective for irregular targets. However, centering prediction may fail when the target’s center is not prominent, potentially leading to missed detections of truck. RetinaNet’s Focal Loss effectively addresses positive-negative sample imbalance in simple backgrounds, but may still require improvement under extremely dense target conditions. For instance, in the fourth row of Figure 13, it consistently detects multiple pedestrians and two trucks. Therefore, without targeted adjustments or the incorporation of multi-feature fusion, Figure 13 demonstrates partial results from the non-visible light universal models FCOS and RetinaNet. Both models exhibit poor overall detection performance.

### 5.4. Ablation Experiments

To quantitatively evaluate the contributions of each architectural component in the MMPFNet framework, we conducted a systematic ablation study. The baseline model used the unmodified YOLOv13n architecture. We first validated the individual contribution of each module, then sequentially integrated them to examine their combined effects, with the training epochs uniformly set to 200. This two-stage ablation design allowed us to independently analyze the impact of each module as well as their progressive integration on both detection accuracy and model complexity.

The ablation study results for the individual contribution of each module are shown in Table 4, while the results for their sequential integration are presented in Table 5. The following observations can be made from these tables:

#### 5.4.1. M-DSC3k2

By employing the M-DSC3k2 module, the model achieves a significant improvement of 8.7% on mAP50-95. This demonstrates that when evaluating metrics with high threshold settings like mAP50-95, the M-DSC3k2 module’s randomly selected attention map captures information across different scales. This enhances the focus on small targets within the dataset, while its contextual learning mechanism also plays a significant role. Simultaneously, this approach also reduces the model’s parameters.

#### 5.4.2. MBConv-Block

Upon incorporating the MBConv-block detection head, the interplay between the MBConv module, the spatial encoding module, and the inverted bottleneck structure within the architecture enables the model to capture richer spatial feature information and achieve deeper feature extraction capabilities while effectively reducing computational complexity. As shown in Table 4, the model equipped with the MBConv-block demonstrates notable gains across all key metrics compared to the baseline. Specifically, the mAP50-95 and Precision increased substantially by 7.4% and 5.0%, respectively. Notably, these gains were achieved alongside a reduction in Parameters and a 17.7% decrease in computational load (GFLOPs). Furthermore, as indicated in the third row of Table 5, the sequential integration of the MBConv-block continues to deliver significant enhancements in mAP50, mAP50-95, Precision, and Recall, while further reducing the computational load from 6.0 GFLOPs to 4.7 GFLOPs—a substantial reduction of 21.7%.

#### 5.4.3. FE-IoU

After introducing FE-IoU, the model incorporates considerations regarding the impact of difficult and easy sample distributions between non-visible light datasets and common visible light datasets on bounding box regression. By focusing on different regression samples, it enhances the detector’s performance across various detection tasks. As demonstrated in the fourth row of Table 4, the model with FE-IoU exhibits notable improvements over the baseline, achieving substantial gains of 8.2% in mAP50-95 and 4.8% in Recall. Furthermore, as shown in the fourth row of Table 5, when FE-IoU is integrated in sequence with other modules, it continues to contribute effectively: while delivering modest improvements in mAP50 and Precision, it also leads to significant increases of 3.0% in mAP50-95 and 3.4% in Recall, respectively.

#### 5.4.4. PPLConv

The PPLConv module is embedded within the primary convolutional network to achieve overall model lightweighting, aiming to adapt the MMPFNet framework for future deployment on resource-constrained edge devices. By simplifying specific convolutional processes in the backbone network, this module significantly reduces model size and computational demands. As shown in the fifth row of Table 4, the standalone integration of PPLConv leads to a substantial reduction in model complexity, with Parameters decreasing by 28.94% and computational load (GFLOPs) dropping by 19.4% compared to the baseline. Although this initial lightweighting comes at a cost, reflected in decreased mAP50, mAP50-95, Precision, and Recall metrics, its subsequent sequential integration yields a more favorable trade-off. Specifically, as demonstrated in the fifth row of Table 5, further incorporating PPLConv into the full module suite continues to deliver significant reductions in both Parameters and GFLOPs, while the corresponding decline in the aforementioned detection accuracy metrics is markedly mitigated. This outcome successfully achieves the intended balance between model accuracy and complexity.

### 5.5. Generalization Experiments

Since MMPFNet was originally designed for target detection in specific non-visible light scenes, we further evaluated its generalization capability. To this end, we constructed an RA dataset from the data packages provided in the CRUW-ONRD dataset and utilized the “single sensor data” package from the “Audio-Visual-Vehicle-Dataset” for soundprint, both of which are applicable to road target detection scenes. MMPFNet, employing a single-frame map input strategy, was trained for 200 epochs on each dataset. The model’s performance is summarized in Table 6. As the table data indicates, MMPFNet maintains high accuracy on these non-visible light datasets while also achieving low parameter counts and GFLOPs consumption.

**Figure 14:**
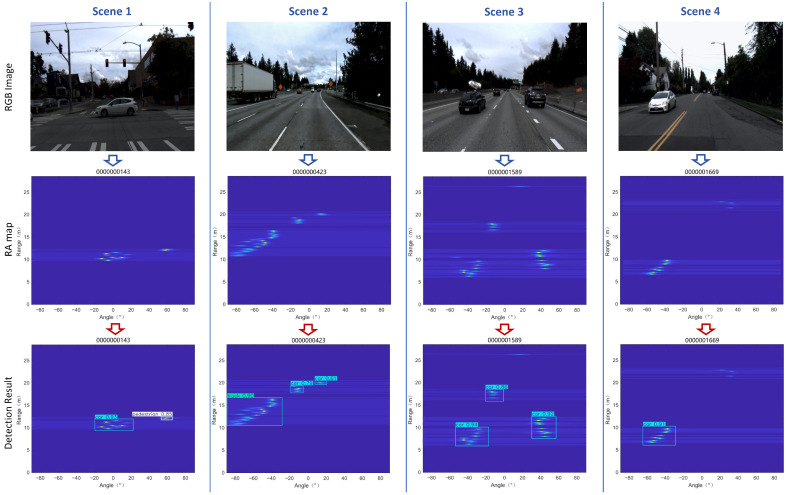
CRUW-ONRD experiment results.

Figure 14 presents the detection results on the CRUW-ONRD dataset. The .npy files from the dataset were converted and processed into RA maps for testing, with detection targets including pedestrians, cars, and trucks. As partially illustrated in Figure 14, MMPFNet accurately identifies these targets, achieving an overall precision of 95.1% (see Table 6 for details). This performance approaches the level achieved on the FMCW RA dataset.

**Figure 15:**
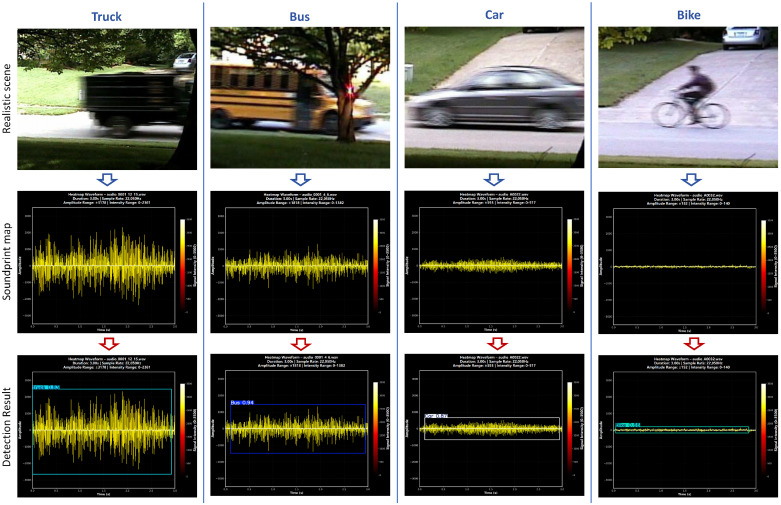
Soundprint experiment results.

During the soundprint experiment, WAV files corresponding to different road targets from the public Audio-Visual-Vehicle-Dataset were converted into soundprint maps using Python 3.9. As illustrated in the second row of Figure 15, these maps encode 3-s audio segments, visualizing information such as amplitude, frequency, and energy distribution in a heatmap format. The corresponding detection results by MMPFNet are displayed in the third row of Figure 15. The model achieves detection precision above 80% for Truck, Bus, and Car targets, yielding an overall precision of 84.4%. However, the detection performance for the Bike class is comparatively lower, which is attributable to its typically lower audio amplitude and limited number of samples in the dataset.

## 6. Discussion

### 6.1. Experimental Analysis

As shown in the experimental results from the previous section, the innovative design of the proposed MMPFNet model is validated. Compared to the baseline, MMPFNet demonstrated significant improvements: mAP50 increased from 93.1% to 97.9%, mAP50-95 rose from 55.4% to 71.4%, precision improved from 92.4% to 98.4%, and recall was enhanced from 87.4% to 96.1%. Simultaneously, it reduces parameters by 45% and GFLOPs by 41.9%. Furthermore, MMPFNet outperforms other common visible-light target detection models in all detection metrics while maintaining lightweight efficiency. Compared to non-visible light detection models, it not only enhances detection accuracy but also significantly reduces missed detection and false detection.

To validate the model’s detection performance on other non-visible light datasets, we conducted generalization experiments using the CRUW-ONRD Dataset and Audio-Visual-Vehicle-Dataset, both featuring traffic scenes. The experimental results demonstrate that MMPFNet also achieves satisfactory detection performance on soundprint and infrared datasets. In summary, MMPFNet achieves an optimal balance between detection accuracy, inference speed, and hardware requirements for road target detection in FMCW radar RA map scenes, while also demonstrating applicability to other scenes.

### 6.2. Limitation and Future Works

The current study focuses on the detection of road targets using single-frame RA maps from a single FMCW radar. While the proposed MMPFNet demonstrates strong performance in this setting, several limitations should be acknowledged.

First, while the experimental evaluation is comprehensive within its defined scope, two major aspects warrant further exploration. Regarding datasets, those used for generalization experiments are not complete or standard benchmarks for millimeter-wave radar detection. They were employed primarily to validate the model’s capability in handling abstract, non-visible light feature representations, thereby demonstrating the architecture’s flexibility. Concerning the comparison with other methods, the evaluation does not encompass several specialized radar detection pipelines (e.g., those based on point clouds, multi-frame tracking, or sensor fusion). This omission stems mainly from the discrepancy between the input data formats required by such methods (e.g., 3D point clouds, temporal sequences, or multimodal data) and the single-frame RA maps format central to this work.

Second, in the generation process of FMCW radar RA maps, there remains room for further improvement in optimizing the detection of transient clutter. Moving forward, we will continue refining the FMCW radar RA map generation process and explore leveraging the collaborative detection capabilities of two FMCW radar RA features to enhance RA dataset quality and broaden the effective detection range. Concurrently, optimizing the antenna weighting coefficient of FMCW radar to improve the accuracy of target data acquisition is also one of our next work directions.

Third, in terms of model optimization, the mAP50-95 metric of the MMPFNet model also holds potential for further enhancement. We plan to investigate more advanced attention mechanisms and loss functions tailored for radar-specific challenges such as multipath interference and low signal-to-noise ratio scenarios.

Finally, in the next phase, we plan to extend MMPFNet by broadening its acceptable input modalities and data formats to support a wider range of representative radar benchmarks, thereby enhancing the system’s applicability. Concurrently, we will undertake comprehensive edge deployment and testing based on MMPFNet to refine its balance between performance and lightweight design. The deployment scheme will systematically integrate an FMCW radar board, a data acquisition board, and a Raspberry Pi to realize a low-cost, lightweight, yet high-precision edge system for real-time road target detection. Through practical implementation and continuous iteration, we aim to further optimize the performance of the MMPFNet-based FMCW radar target detection system, providing insightful references for the broader field of road target perception.

## 7. Conclusions

This paper focuses on road target detection using a single FMCW radar. By converting road target data collected by the FMCW radar into RA video, each frame of the RA map undergoes NCI, SA-CFAR, peak search, and MUSIC super-resolution processing. This generates an optimized FMCW radar RA map dataset. Given the challenges posed by the FMCW radar RA map dataset—characterized by non-visible light, abstract representations, and the difficulty of detecting small targets that are prone to overlap—we propose the M-DSC3k2 module. This module employs MCAttn embedding with depth-separable convolutions to enhance attention for small target detection. We design a detection head with a dual-bottleneck inverted MBConv-block structure to improve the model’s accuracy and generalization capabilities. A lightweight PPLConv module is customized to modify the backbone network, achieving significant model weight reduction while only slightly compromising accuracy. Considering the distinct characteristics of the target dataset compared with traditional visible-light datasets, we introduce the FE-IoU loss function to focus on different regression samples. Ultimately, the attention-enhanced lightweight model MMPFNet based on hypergraph mechanisms is obtained. The model demonstrates significant improvements over the baseline in key evaluation metrics including mAP50, mAP50-95, precision, and recall. It also achieves a more lightweight design, substantially reducing both the number of parameters and GFLOPs. Furthermore, MMPFNet exhibits strong performance on other public non-visible light datasets.

## Figures and Tables

**Table 1:** Experimental Evaluation Metrics Statistics for MMPFNet.

Model	mAP50	mAP50-95	Precision	Recall	Parameters	GFLOPs
YOLOv13n	93.1%	55.4%	92.4%	87.4%	2.45M	6.2
MMPFNet	97.9%	71.4%	98.4%	96.1%	1.35M	3.6

**Table 2:** Comparison of Several Common General Model Metrics.

Metric	Faster R-CNN	RT-DETR	YOLOv5n	YOLOv8n	YOLOv11n	YOLOv12n	YOLOv13n	MMPFNet
mAP50	80.0%	90.5%	89.4%	90.7%	90.2%	89.5%	93.1%	97.9%
mAP50-95	68.0%	56.9%	55.8%	54.3%	53.6%	51.5%	55.4%	71.4%
Precision	85.0%	87.1%	86.0%	89.1%	88.3%	89.3%	92.4%	98.4%
Recall	80.2%	89.3%	84.4%	84.2%	85.4%	86.4%	87.4%	96.1%
Parameters	3,019,039	31,991,960	2,503,724	3,006,428	2,582,932	2,509,124	2,448,675	1,346,211
GFLOPs	15.5	103.4	7.1	8.1	6.3	5.8	6.2	3.6

**Table 3:** Detection Parameter Comparison of MMPFNet, FCOS, and RetinaNet on CRUW-ONRD and FMCW Radar RA Map Datasets.

Dataset	Model	Backbone	mAP50	mAP50-95	Precision	Recall
CRUW-ONRD	FCOS	ResNet-101	81.90%	51.30%	77.10%	82.20%
	RetinaNet	ResNet-101	77.40%	48.90%	75.50%	79.30%
	MMPFNet	–	94.50%	70.80%	95.10%	94.20%
FMCW RA	FCOS	ResNet-101	85.20%	55.10%	80.60%	86.50%
	RetinaNet	ResNet-101	82.50%	52.20%	77.20%	83.50%
	MMPFNet	–	97.90%	71.40%	98.40%	96.10%

**Table 4:** Ablation Study on the Individual Contribution of Each Module.

M-DSC3k2	MBConv-Block	FE-LoU	PPLConv	mAP50	mAP50-95	Precision	Recall	Parameters	GFLOPs
×	×	×	×	93.1%	55.4%	92.4%	87.4%	2,448,675	6.2
✓	×	×	×	95.7%	64.1%	93.5%	91.0%	2,030,563	6.0
×	✓	×	×	95.4%	62.8%	97.4%	91.8%	2,140,051	5.1
×	×	✓	×	96.3%	63.6%	93.3%	92.2%	2,448,675	6.2
×	×	×	✓	92.2%	53.9%	91.5%	86.1%	1,764,483	5.0
✓	✓	✓	✓	97.9%	71.4%	98.4%	96.1%	1,346,211	3.6

**Table 5:** Ablation study on the Sequential Integration of Modules.

M-DSC3k2	MBConv-block	FE-IoU	PPLConv	mAP50	mAP50-95	Precision	Recall	Parameters	GFLOPs
×	×	×	×	93.1%	55.4%	92.4%	87.4%	2,448,675	6.2
✓	×	×	×	95.7%	64.1%	93.5%	91.0%	2,030,563	6.0
✓	✓	×	×	97.6%	69.4%	98.3%	94.9%	2,030,691	4.7
✓	✓	✓	×	98.4%	72.4%	98.9%	97.3%	2,030,691	4.7
✓	✓	✓	✓	97.9%	71.4%	98.4%	96.1%	1,346,211	3.6

**Table 6:** MMPFNet Generalization Experiment Results.

Dataset	mAP50	mAP50-95	Precision	Recall	Parameters	GFLOPs
FMCW RA Dataset	97.9%	71.4%	98.4%	96.1%	1,346,211	3.6
CRUW-ONRD Dataset	94.5%	70.8%	95.1%	94.2%	1,360,748	3.6
Audio-Visual Vehicle Dataset	90.8%	64.4%	84.4%	91.0%	1,350,563	3.5

## Data Availability

The original datasets presented in this study are available on request from the corresponding author.
